# Supplementary figures and images for: Ultrasound and Clinical Preoperative Characteristics for Discrimination Between Ovarian Metastatic Colorectal Cancer and Primary Ovarian Cancer: A Case-Control Study
Source: Diagnostics (Basel). 2019 Dec 1;9(4):210. doi: 10.3390/diagnostics9040210 (PMC6963303; doi:10.3390/diagnostics9040210)

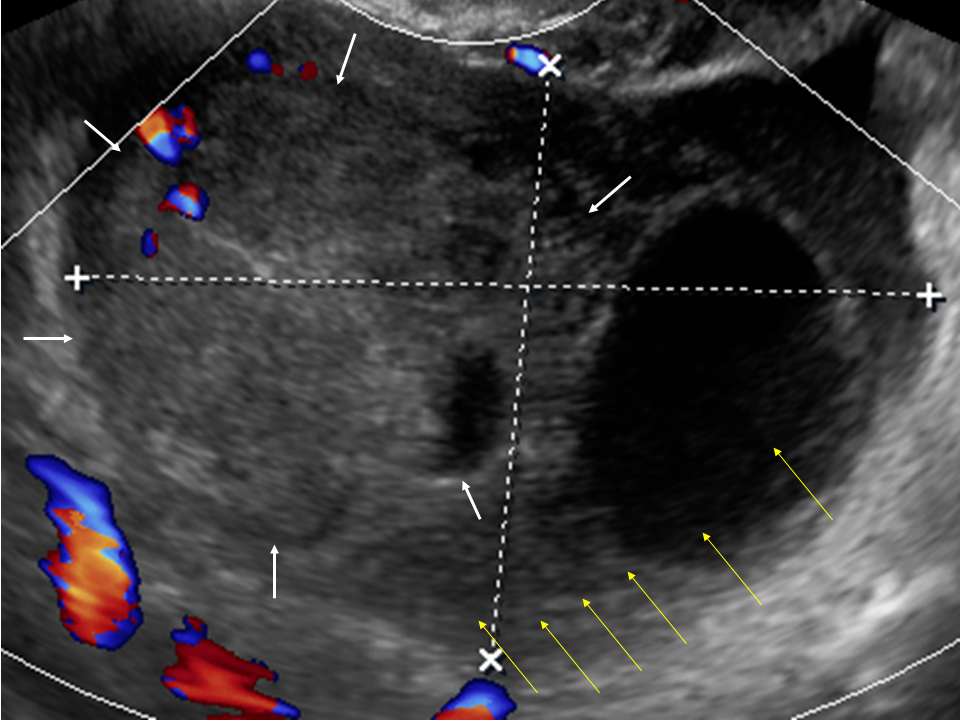

Supplement: Supplementary file 1 [file diagnostics-09-00210-s001.zip › Figure S1.tif]

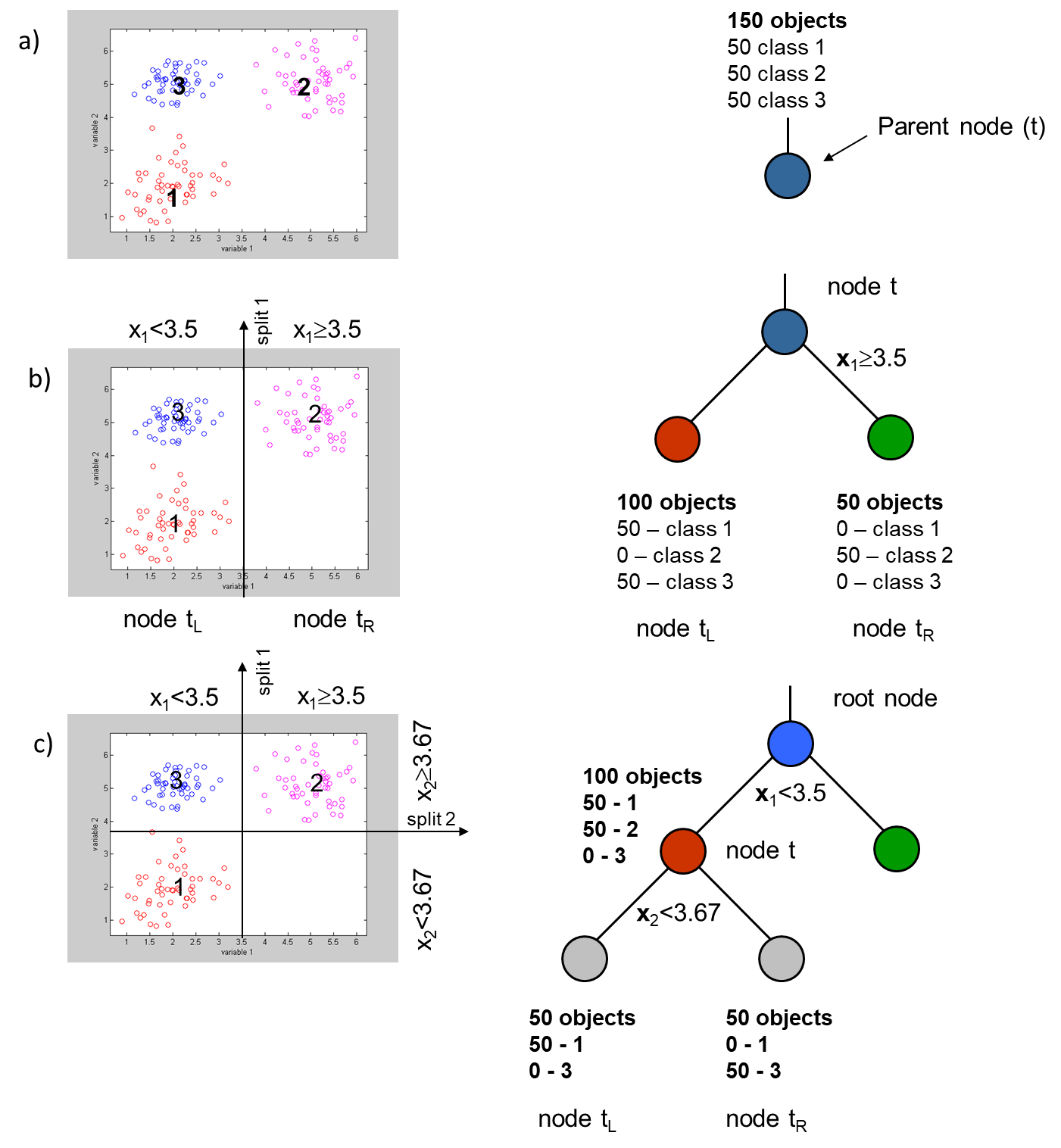

Supplement: Supplementary file 1 [file diagnostics-09-00210-s001.zip › Figure S2.tif]

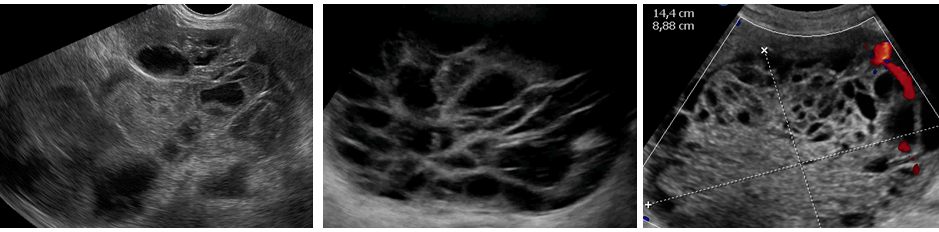

Supplement: Supplementary file 1 [file diagnostics-09-00210-s001.zip › Figure S3.tif]

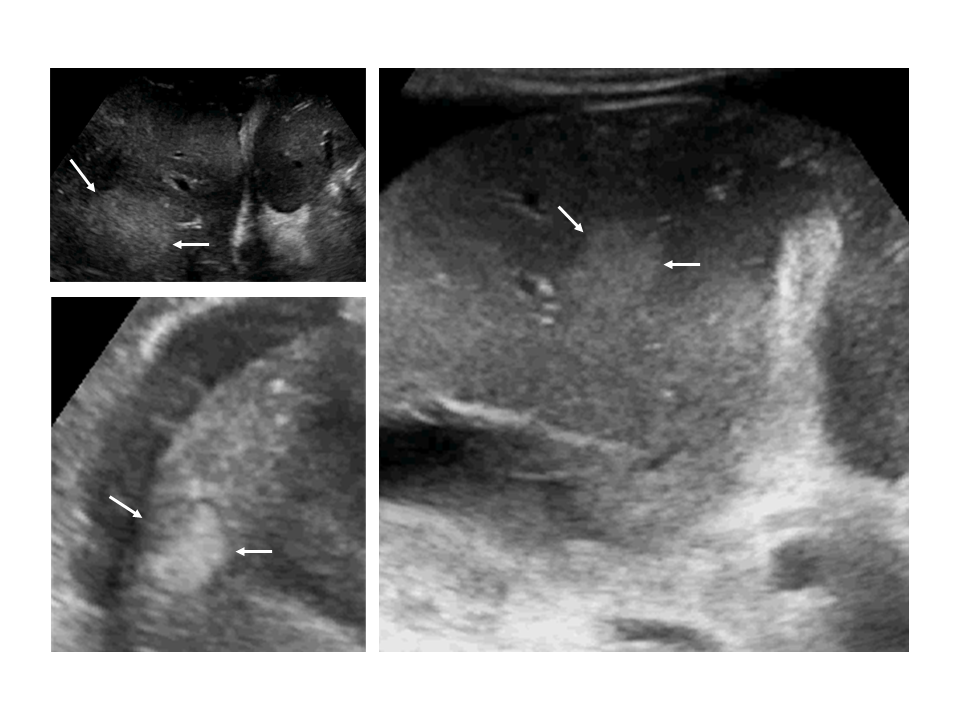

Supplement: Supplementary file 1 [file diagnostics-09-00210-s001.zip › Figure S4.tif]

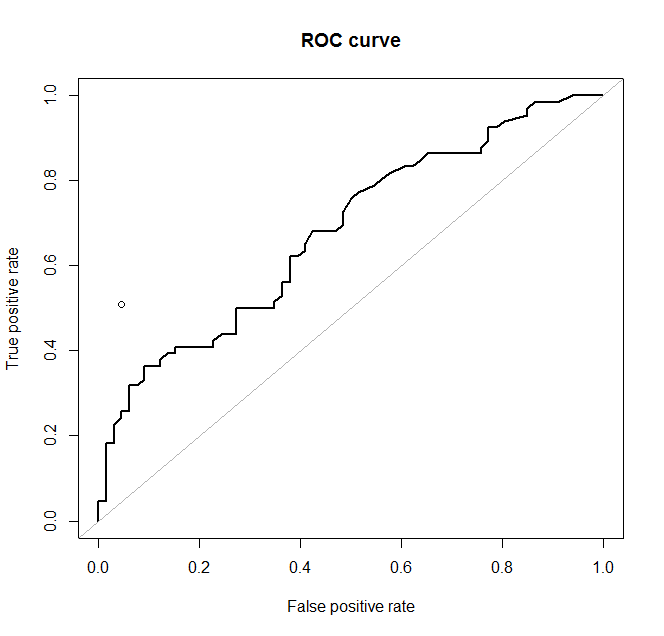

Supplement: Supplementary file 1 [file diagnostics-09-00210-s001.zip › Figure S5.tif]

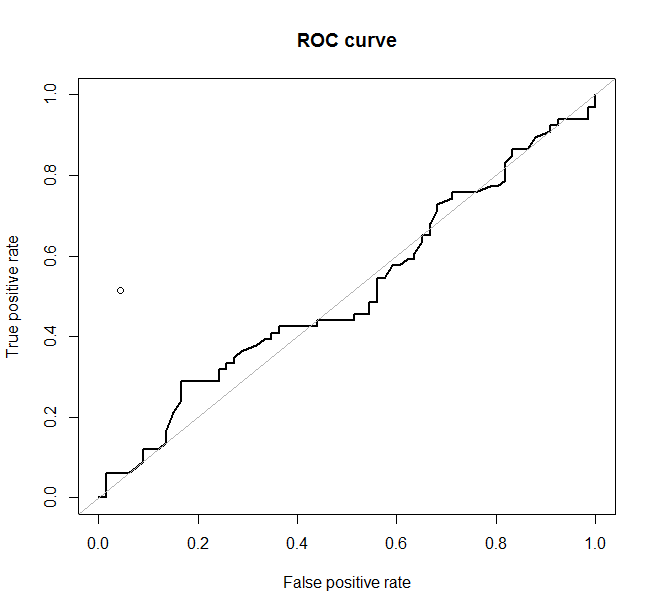

Supplement: Supplementary file 1 [file diagnostics-09-00210-s001.zip › Figure S6.tif]
